# The Royal Medical Benevolent Fund Guild

**Published:** 1920-11-13

**Authors:** 


### The Royal Medical Benevolent Fund Guild.

The Royal Medical Benevolent Fund is an old-established institution for the aged of the medical profession who can no longer support themselves, and the necessitous relatives of those medical men who, in the practice of their profession, have lost their health or their lives before they have been able to make adequate provision for their dependants.

In common with other charities the Guild has been "hard hit" by the war, and a determined attempt is now being made to put its finances on a sound basis, and a sum of £10,000 is needed for the educational branch alone.

Assistance is solicited for (*a*) personal service, (*b*) providing clothing, (*c*) giving other help as recommended by the General Purposes Committee of the Guild, (*d*) promoting any other work in connection with the Guild which may from time to time seem desirable and be approved by the Executive Committee of the Fund. During the last few years scholarships have been founded to educate a number of boys for the great professions, thus giving them a chance to continue in their fathers' sphere of life, and not to drift into the more nebular and more hazardous occupations.

In order to help this excellent and necessary work a special matinée will be held on Friday, November 19, at His Majesty's Theatre, when a number of famous actors and actresses will perform. Seldom have so many distinguished artistes been associated together on a stage at one performance.

Amongst those who have promised to give their services are Miss Gladys Cooper, Miss Fay Compton, Miss Winifred Emery, and Messrs. Nelson Keys, Gerald du Maurier, Basil Rathbone, Owen Nares, Leslie Faber, George Grossmith, Godfrey Tearle, Leslie Henson, and Tom Walls. Prices of seats will range from 1s. 3d. to £3 3s.

Tickets may be obtained from Lady Fripp, 19 Portland Place, W. 1.

H.R.H. Princess Christian has signified her intention of being present.

